# Cytokine Receptor-Like Factor 3 (CRLF3) Contributes to Early Zebrafish Hematopoiesis

**DOI:** 10.3389/fimmu.2022.910428

**Published:** 2022-06-20

**Authors:** Tarannum Taznin, Kaushalya Perera, Yann Gibert, Alister C. Ward, Clifford Liongue

**Affiliations:** ^1^ School of Medicine, Deakin University, Geelong, VIC, Australia; ^2^ Institute for Mental and Physical Health and Clinical Translation, Deakin University, Geelong, VIC, Australia

**Keywords:** CRLF3, hematopoiesis, cytokine receptor, zebrafish, embryogenesis

## Abstract

Cytokine receptor-like factor 3 (CRLF3) is an ancient protein conserved across metazoans that contains an archetypal cytokine receptor homology domain (CHD). This domain is found in cytokine receptors present in bilateria, including higher vertebrates, that play key roles in a variety of developmental and homeostatic processes, particularly relating to blood and immune cells. However, understanding of CRLF3 itself remains very limited. This study aimed to investigate this evolutionarily significant protein by studying its embryonic expression and function in early development, particularly of blood and immune cells, using zebrafish as a model. Expression of *crlf3* was identified in mesoderm-derived tissues in early zebrafish embryos, including the somitic mesoderm and both anterior and posterior lateral plate mesoderm. Later expression was observed in the thymus, brain, retina and exocrine pancreas. Zebrafish *crlf3* mutants generated by genome editing technology exhibited a significant reduction in primitive hematopoiesis and early definitive hematopoiesis, with decreased early progenitors impacting on multiple lineages. No other obvious phenotypes were observed in the *crlf3* mutants.

## Introduction

Cytokines and their specific receptors represent key components of cell-to-cell communication in multicellular organisms (1). Together they play significant roles in the production and function of blood and immune cells as well as other developmental and homeostatic processes (2). Cytokine receptors are complex proteins with a number of different domains, but all share a cytokine receptor homology domain (CHD) that consists of two fibronectin (FBN) type III folds bearing one or two paired cysteine residues with or without a WSXWS motif depending on whether it is a class I or class II receptor (1). Higher vertebrates possess a large family of cytokine receptors that contain one or more class I-type CHD, along with additional extracellular domains as well as a transmembrane and intracellular region, as well as a variety of receptors containing class II type CHDs which play important roles particularly in blood and immune cell development (3). Cytokine receptors arose in bilateria with the emergence of a protein related to the higher vertebrate GP130, typified by Domeless in fruit fly (*Drosophila melanogaster*) that acts as a classical cytokine receptor (3). All use the Janus kinase-Signal transducer and activator of transcription (JAK-STAT) pathway to mediate intracellular signaling (1).

Cytokine receptor like factor 3 (CRLF3) consists mostly of a class I CHD (3, 4). This ancient protein has been found across metazoan, suggesting it is the likely precursor of this important structural element of cytokine receptors (5), but is also retained in bilateria, including high vertebrate species (3). However, in stark contrast to cytokine receptors, little is known about CRLF3 and its function. Available data indicate broad expression in mice (6) and humans (7), including hematopoietic organs in both cases (6, 7). The limited functional studies have suggested potential neuronal functions in invertebrates (8, 9) and higher vertebrates (10). The invertebrate studies have also suggested that CRLF3 is activated by unknown cytokines (9) and utilizes the JAK-STAT pathway (8), although the details remain scant.

Vertebrate hematopoiesis occurs *via* a conserved program of development involving distinct waves (11). Primitive hematopoiesis in zebrafish commences in the anterior lateral mesoderm (ALM) and posterior lateral mesoderm (PLM) from precursors expressing *scl* that contribute to both blood and vascular development (12). Primitive myeloid cells are largely derived from *spi1*-expressing cells in the ALM whereas primitive erythrocytes develop from *gata1*-expressing cells in the PLM (13, 14), with a transient intermediate wave in the posterior blood island (PBI) contributing to both erythrocytes and granulocytes (15). Definitive hematopoiesis commences with the generation of multipotent hematopoietic stem cells (HSCs) expressing *cmyb* in the ventral wall of the dorsal aorta within the aorta-gonad-mesonephros region (16) and migrate first to the caudal hematopoietic tissue (CHT), equivalent to the mammalian fetal liver and then the kidney marrow, equivalent to the mammalian bone marrow, to generate multiple blood lineages (12, 17, 18). Lymphocyte progenitors expressing *ikzf1* populate the zebrafish thymus early in embryogenesis with T lymphopoiesis well established by 5 dpf (19). Zebrafish B lymphocytes develop around 20 dpf (18), with NK-related cells evident at this time (20). Conserved cytokine receptor homologues have been shown to be expressed in and contribute to the development of blood and immune cells, including erythropoietin receptor (EPO-R) for erythrocytes (21), granulocyte colony-stimulating factor receptor (GCSF-R) for granulocytes (22) and interleukin 2 receptor (IL-2R) family members for T lymphocytes (23).

This study aimed to use zebrafish as a model to further investigate CRLF3. This involved analysis of its spatio-temporal expression pattern and employing genome editing to assess the impact of its ablation during early development, with an emphasis on hematopoiesis.

## Materials and Methods

### Zebrafish Husbandry

Wild-type and mutant zebrafish were maintained using standard husbandry practices (24). This included feeding thrice daily with a mixture of live feed (artemia and rotifers) and a dry granulated foodstuff (Otohime Hirame Japan). Fish were manually spawned and embryos maintained in a petri dish containing aquarium water with 0.00005% (w/v) methylene blue (Sigma-Aldrich) and raised in an incubator at 28.5°C with 0.03% (w/v) 1-phenyl-2-thio-urea (PTU) used from 8 h post fertilization (hpf) to inhibit pigmentation to enhance embryo transparency. All experiments were approved by the Deakin University Animal Welfare Committee.

### Embryo Analysis

At appropriate time points embryos were collected and anesthetized with 0.4 mg/mL benzocaine and finally fixed with 4% (w/v) paraformaldehyde (PFA) made in phosphate-buffered saline (PBS). Fixed embryos were subjected to whole-mount *in situ* hybridization (WISH) with DIG-labeled probes as described (25). To make probes, 1-2 µg linearized DNA or 100-500 ng PCR products were incubated in DIG labelling mix (Roche) with 20 U RNase inhibitor and 40 U T7 or SP6 RNA polymerase. Following incubation at 37°C for 2 h, 20 U DNase I was added and the sample incubated at 37°C for a further 15 min before addition of EDTA (pH 8.0) to 20 mM, with the probe purified using G-50 gel filtration exclusion microcolumns (GE Health). Fixed embryos were rehydrated *via* a series of sequential 5 min washes in 75% (v/v) methanol, 50% (v/v) methanol, 25% (v/v) methanol and PBS before 4 × 5 min washes in PBS containing 0.1% Tween-20 (PBS-T). Embryos were then incubated at room temperature in PBS-T containing 10 µg/mL proteinase K for 1-30 min before fixing for 20 min in 4% (v/v) PFA/PBS followed by 5 × 5 min washes in PBS-T. Embryos were incubated in hybridization mix (HM) (50% formamide, 5×SSC, 0.1% Tween-20, 9.2 mM citric acid pH 6, 50 µg/mL heparin, 500 µg/mL tRNA) for 2-5 h at 70°C. Embryos were then bathed in probe solution (1/50–1/300 dilution of DIG-labelled RNA in HM+) and incubated at 70°C overnight. Probe solution was removed and embryos washed briefly in 100% HM– (50% formamide, 5×SSC, 0.1% Tween-20, 9.2 mM citric acid pH 6) and then sequentially for 15 min in 75% HM–/25% 2×SSC, 50% HM–/50% 2×SSC, 25% HM–/75% 2×SSC and 2×SSC, then twice in 0.2×SSC 30 min, then sequentially for 10 min in 75% 0.2×SSC/25% PBS-T, 50% 0.2×SSC/50% PBS-T, 25% 0.2×SSC/75% PBS-T and PBS-T. Embryos were then incubated in PBS-T containing 2% sheep serum and 2 mg/mL BSA at room temperature for 3 h then in antibody solution (1:5000 anti-DIG antibody in PBS-T, 2% sheep serum, 2 mg/mL BSA pre-absorbed against embryos) at 4°C overnight with agitation. Embryos were rinsed in PBS-T then 6 × 15 min washes in PBS-T at room temperature with gentle rocking and then 3 × 5 min washes in staining buffer (0.1 M Tris pH 9.5, 0.05 M MgCl_2_, 0.1 M NaCl, 0.1% (v/v) Tween 20) containing 25 µg/mL levamisol. Following this, embryos placed in staining buffer containing 0.225 mg/mL nitroblue tetrazolium (NBT) and 90.175 mg/mL 5-bromo,4-chloro,3-indolyl phosphate (BCIP) at room temperature in the dark for 4 h-3 d. Embryos were finally rinsed in PBS-T and fixed in 4% PFA/PBS when stained to an appropriate extent. In some experiments, embryos were injected at the 1-4 cell stage with standard control or *lycat* morpholinos as described (15), or bathed in 30 µM JAK3 inhibitor or DMSO as a control as published (26). Embryos were visualized using a MVX10 microscope (Olympus) with stage lighting provided using an LG-PS2 fibre optics light source (Olympus). Digital images were recorded using CellSens Dimension 1.6 software (Olympus) and DP72 camera (Olympus). Alternatively, cytospin preparations were prepared from embryonic blood, as described (27). These were fixed in 100% methanol for 1 min and stained with Giemsa for 20 min. Slides were viewed on a Leica DME stereomicroscope and differential counts performed, with images captured on a DFC290 digital camera (Leica).

### Genome Editing

The zebrafish *crlf3* gene was targeted using genome editing with transcription activator-like effector nucleases (TALENs) and CRISPR/Cas9 (28). A guide RNA (gRNA) was designed to a region of exon 2 using the zifit protocol (29) with the primers 5’-TAGGCAGAGGCGTTGCTGCAGG and 5’-AAACCCTGCAGCAACGCCTCTG, while a pair of TALENs targeting a similar region were designed as described (30). Embryos were injected with 12.5 ng/μL gRNA and 300 ng/μL Cas9 mRNA or with 100 ng/μL mRNA encoding each TALEN and raised to adulthood. Founders were identified with high-resolution melt (HRM) analysis of PCR products with Precision Melt Suremix and Analysis Software (BioRad) (31) using primers spanning the targeted region (5’- CTATTTAGCAGCATGAGTTTACAGC and 5’-TAACAAGGGTTTCTGACTTCTATGC). These fish were outcrossed two times to wild-type fish to remove off-target mutations before in-crossing. Sequence analysis was performed at the Australian Genome Research Centre.

### Statistics

Statistical analyses were performed using Graph Pad Prism (Version 8) software. To determine the statistical significance of various treatments, the unpaired independent student’s *t* test was employed, with Welch’s correction, where appropriate.

### Bioinformatics

Protein sequences obtained from GenBank were aligned with Clustal Omega (version 1.2.4) (32), with a phylogenetic tree generated with NJ Plot (33).

## Results

### Expression of Zebrafish *crlf3*

**Figure 1:**
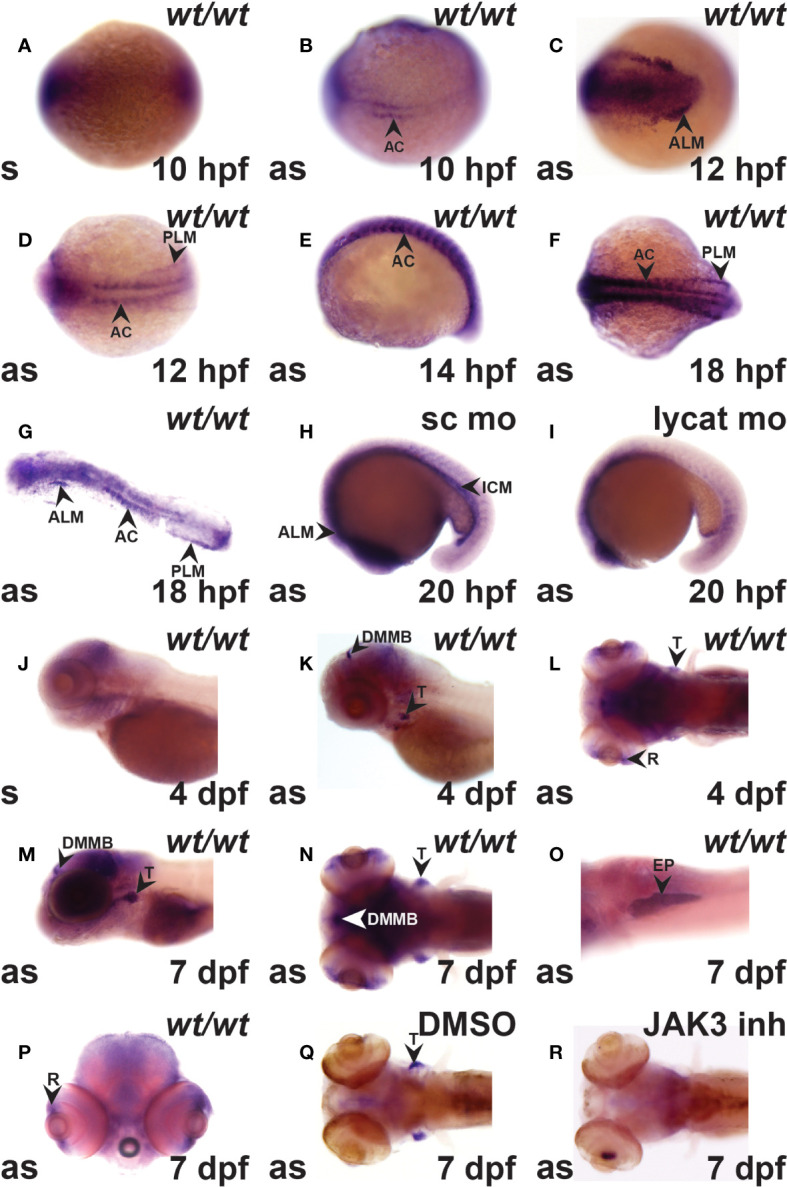
The *crlf3* gene is expressed in hematopoietic and other tissues during zebrafish embryogenesis. **(A–R)**. WISH with sense (s) or antisense (as) *crlf3* probes at the indicated time points on wild-type embryos (*wt/wt*, **A–G, J–P**) or those injected with 1 mM standard control morpholino (sc mo, **H**) or *lycat* morpholino (lycat mo, **I**) or bathed from 56 hpf in DMSO vehicle control (DMSO, **Q**) or JAK3 inhibitor (JAK3 inh, **R**). Embryos are dorsal view with anterior to the left **(A, B, D, F, L, N, Q, R)**; anterior view with dorsal to the left **(C)**; lateral view with anterior to the left **(E, J, K, M)**, except panel O that is ventral view with anterior to the left and P is anterior view with dorsal to the top. The indicated structures are: AC (adaxial cells), ALM (anterior lateral plate mesoderm), DMMB (dorsal midline of midbrain), EP (exocrine pancreas), ICM (intermediate cell mass), PLM (posterior lateral plate mesoderm), R (retina), and T (thymus).

Expression of *crlf3* during zebrafish embryogenesis was examined by WISH with a full-length anti-sense probe in comparison to a control sense probe. The sense probe gave a small amount of diffuse background staining (**Figures 1A, J**). However, the anti-sense probe identified specific *crlf3* expression, first evident from 10 hpf in adaxial cells in the presomitic mesoderm (**Figure 1B**) that continued until 18 hpf before waning (**Figures 1D–G**). Bilateral expression was observed from 12 hpf in the anterior lateral-plate mesoderm (ALP) (**Figure 1C**) and posterior lateral-plate mesoderm (PLM) (**Figure 1D**), which continued beyond 20 hpf when the PLM fuses to form the intermediate cell mass (ICM) (**Figures 1F–H**). To confirm this designation embryos were injected with a *lycat* antisense morpholino to ablate both hematopoietic and endothelial lineages in the ICM (34), which also greatly diminished *crlf3* expression in this region (**Figure 1I**) compared to those injected with standard control morpholino (**Figure 1H**). By 4 dpf, *crlf3* expression was observed in the thymus, dorsal midline of the midbrain and retina (**Figures 1K–L**), and from 7 dpf expression become stronger with additional expression observed in the exocrine pancreas and retina (**Figures 1M–P**). Embryos treated with a JAK3 inhibitor (35) that is able to ablate T lymphocytes in zebrafish (26) showed no *crlf3* expression in the thymus (**Figure 1R**), while those treated with DMSO only exhibited robust expression (**Figure 1Q**).

### Generation of *crlf3* Knockout Zebrafish

**Figure 2:**
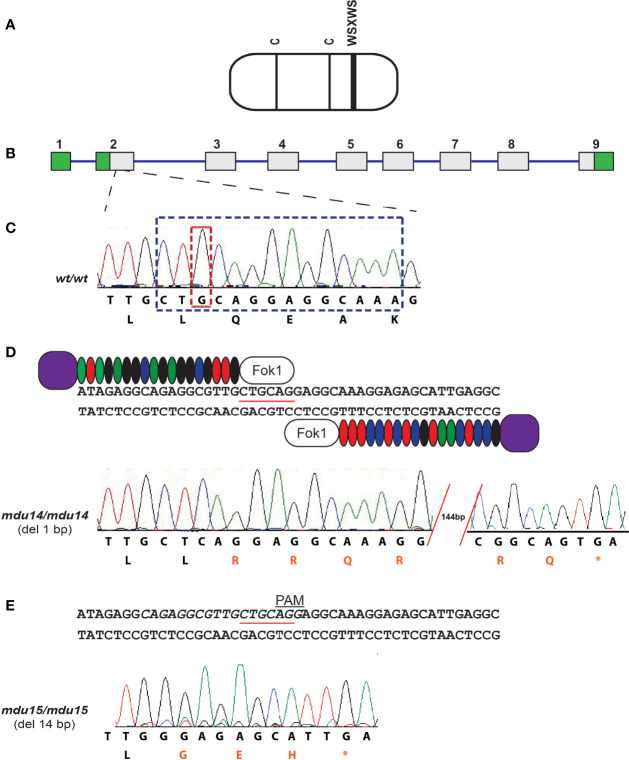
Generation of zebrafish *crlf3* mutants using genome editing. **(A)** Schematic representation of the CRLF3 protein, consisting mostly of a cytokine receptor homology domain (rounded rectangle) containing two conserved cysteines (**C**, thin lines) and a WSXWS motif (thick line). **(B)** The intron/exon structure of the zebrafish *crlf3* gene, with exons represented as numbered boxes, showing untranslated (green) and translated (gray) regions, and introns represented with intervening lines. **(C)** Sequence trace of homozygous wild-type *crlf3^wt/wt^* (*wt/wt*) and its corresponding nucleotide sequence and encoded amino acids shown below. Nucleotides deleted in *mdu14* and *mdu15* alleles are boxed in tan and blue, respectively. **(D)** Targeting of exon 2 with TALENs, with the *Pst*I site used in RFLP analysis underlined, to generate the *mdu14* allele, with the sequence of a homozygous *crlf3^mdu14/mdu14^* (*mdu14/mdu14*) mutant shown. This represents a 1 bp deletion that causes a frameshift resulting in translation from an alternative reading frame (red) followed by a stop codon (*) that prematurely truncates the protein. **(E)** Targeting of exon 2 with CRISPR, with target site italicized and PAM site indicated, to generate the *mdu15* allele, with the sequence of a homozygous *crlf3^mdu15/mdu15^* (*mdu15/mdu15*) mutant shown. This 14 bp deletion also causes a frameshift and premature stop.

The zebrafish Crlf3 protein (**Figure 2A**) is encoded by a gene that consists of nine exons and eight introns (**Figure 2B**). To study the role of *crlf3* both TALEN (**Figure 2D**) and CRISPR-Cas9 (**Figure 2E**) based genome editing approaches were designed to target adjacent sites in the coding region of exon 2 of the wild-type (*wt*) gene (**Figure 2C**). One-cell stage embryos were injected with either TALEN mRNA or Cas9 mRNA plus a guide RNA and raised to adulthood and crossed with wild-type embryos, with their progeny analyzed by high-resolution melt analysis and sequencing to identify potential heterozygote mutants. After a further round of out-crossing, heterozygote F2 mutants were in-crossed to yield homozygote F3 mutants. Sequencing identified two mutant alleles: a TALEN derived 1 bp deletion, designated *mdu14* (**Figure 2D**), and a CRISPR-Cas9 derived 14 bp deletion, designated *mdu15* (**Figure 2E**). Both of these mutations serve to severely truncate the encoded Crlf3 protein after just 8 or 7 residues, respectively, due to a frame-shift followed by an in-frame stop codon (**Figure 2D, E**), and so likely represent loss-of-function alleles.

### Impact of *crlf3* Ablation on Primitive Hematopoiesis

**Figure 3:**
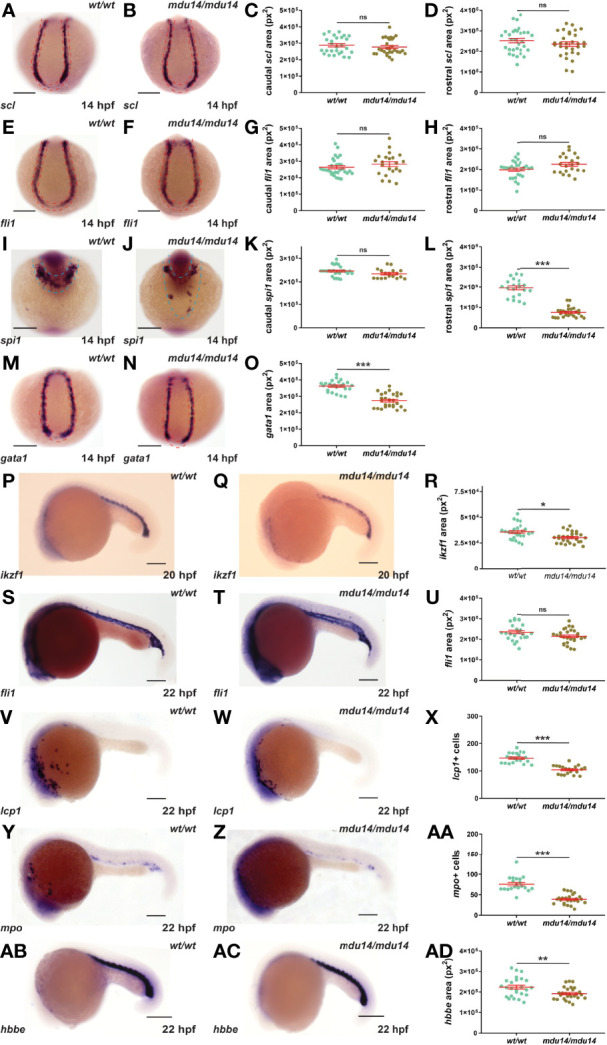
Mutation of *crlf3* impacts primitive hematopoiesis. **(A–AD)**. Homozygous *crlf3^wt/wt^* (*wt/wt*), and *crlf3^mdu14/mdu14^* (*mdu14/mdu14*) embryos were subjected to WISH at 14 hpf with *scl* **(A, B)**, *fli1* **(E, F)**, *spi1* **(I, J)** and *gata1* **(M, N)**, at 20 hpf with *ikzf1* **(P, Q)**, and at 22 hpf with *fli1* **(S, T)**, *lcp1* **(V, W)**, *mpo* **(Y, Z)** and *hbbe* **(AB, AC)** (scale bar = 200 μm; red and blue dotted areas depict caudal and rostral expression domains, respectively). Individual embryos were assessed for area of staining or cell number at the indicated locations for *scl* **(C, D)**, *fli1* **(G, H, U)**, *spi1* **(K, L)**, *gata1* **(O)**, *ikzf1* **(R)**, *lcp1* **(X)**, *mpo* **(AA)** and *hbbe* **(AD)**, with the mean and SEM shown in red and level of statistical-significance indicated (***p < 0.001, **p < 0.01, *p < 0.05, ns, not significant). Welch’s correction was used for panel **(R)**.

No evidence of overt developmental perturbation during embryogenesis was observed in *crlf3* mutants by light microscopy. However, given the strong expression of *crlf3* at the sites of embryonic blood and immune cell development, and the extensive involvement of cytokine receptors in these lineages, this was explored in more detail. The *crlf3* mutants were first analyzed for primitive hematopoiesis using WISH. At 14 hpf, *crlf3^mdu14/mdu14^* mutants and their wild-type siblings showed equivalent expression of *scl*, a marker of hemangioblasts (36), both caudally and rostrally (**Figures 3A–D**), and of *fli1*, a marker of vascular precursors (36), both caudally and rostrally (**Figures 3E–H**). In contrast, decreased expression for *spi1*, a marker of myeloid precursors (37, 38), was observed in *crlf3^mdu14/mdu14^* embryos that reached significance for the rostral domain (**Figures 3I–L**), as well as for *gata1*, a marker erythroid precursors expressed solely in the caudal region of the embryo (39) (**Figures 3M–O**). Expression of *ikzf1*, which marks hematopoietic progenitors during primitive hematopoiesis (40), was also decreased at 20 hpf (**Figures 3P–R**), whereas expression of *fli1* remained unchanged at 22 hpf (**Figure 3S–U**). However, *crlf3^mdu14/mdu14^* embryos showed decreased numbers of cells expressing *lcp1* (**Figures 3V–X**) and *mpo* (**Figure 3Y–AA**), markers of monocyte/macrophages (41) and neutrophils (13), respectively. In addition, a more subtle but still statistically significant decrease in expression was observed for *hbbe* (**Figures 3AB–AD**), a marker of mature erythroid cells (42). Analysis of *crlf3^mdu15/mdu15^* mutants confirmed the results for *ikzf1* (**Supplementary Figures 1A–C**), *lcp1* (**Supplementary Figure 1D**), *mpo* (**Supplementary Figure 1E**) and *hbbe* (**Supplementary Figure 1F**).

### Impact of *crlf3* Ablation on Early Definitive Hematopoiesis

**Figure 4:**
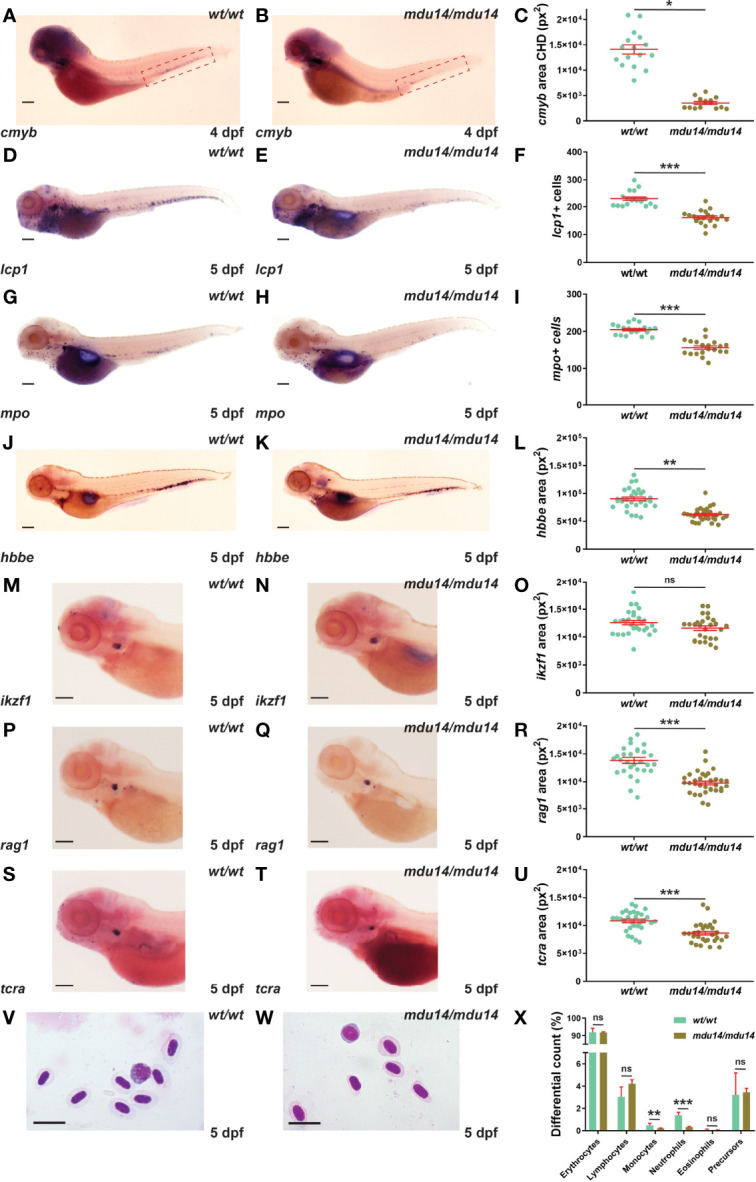
Mutation of *crlf3* impacts early definitive hematopoiesis. **(A–X)**. Homozygous wild-type *crlf3^wt/wt^* (*wt/wt*) and *crlf3^mdu14/mdu14^* (*mdu14/mdu14*) embryos were subjected to WISH with *cmyb* **(A, B)** at 4 dpf, and *lcp1* **(D, E)**, *mpo* **(G, H)**, *hbbe* **(J, K)**, *ikzf1* **(M–N)**, *rag1* **(P, Q)** and *tcra* **(S, T)** at 5 dpf (scale bar = 200 μm), or underwent blood analysis **(V–W)** at 5 dpf (scale bar = 10 μm). Individual embryos were assessed for the area of staining of *cmyb* CHD region (dotted boxes in panels **A** and **B**) **(C)**, *hbbe* **(L)**, *ikzf1* **(O)**, *rag1* **(R)** and *tcra* **(U)**, as well as the number of *lcp1^+^* **(F)** and *mpo^+^* **(L)** cells or for blood differential counts **(X)**, with mean and SEM in red and statistical significance indicated (***p < 0.001, **p < 0.01, *p < 0.05, ns, not significant). Welch’s correction was used for panels **(C**, **L)**.

The *crlf3* mutants were also examined with respect to early definitive hematopoiesis, which generates both myeloid and lymphoid cells from around 2-3 dpf (43). Decreased expression of *cmyb*, a marker of hematopoietic stem cells in the caudal hematopoietic tissue (16), was observed in *crlf3^mdu14/mdu14^* embryos at 4 dpf (**Figures 4A–C**). This correlated with a substantial reduction in the number of cells expressing *lcp1* in (**Figures 4D–F**) and of those expressing *mpo* (**Figures 4G–I**) at 5 dpf. The *crlf3^mdu14/mdu14^* embryos also showed decreased expression of *hbbe* (**Figures 4J–L**). Expression of *ikzf1*, a marker of lymphocyte precursors in the thymus (40), was not significantly different between *crlf3^mdu14/mdu14^* embryos and their wild-type counterparts (*p* = 0.083) (**Figures 4M–O**). However, a small decrease was seen in the expression of two markers of mature lymphoid cells, *rag1* (44) (*p* < 0.001) (**Figures 4P–R**) and *tcra* (45) (*p* < 0.001) (**Figures 4S–U**). Analysis of blood smears from 5 dpf embryos identified a decrease in circulating monocytes (*p* = 0.022) and neutrophils (*p* < 0.001) (**Figures 4V–X**), highlighting that these populations were particularly impacted. Analysis of *crlf3^mdu15/mdu15^* mutants confirmed the results for *cmyb* (**Supplementary Figures 1G–I**), *lcp1* (**Supplementary Figure 1J**), *mpo* (**Supplementary Figure 1K**) and *hbbe* (**Supplementary Figure 1L**).

### Impact of *crlf3* Ablation on Other Aspects of Embryogenesis

**Figure 5:**
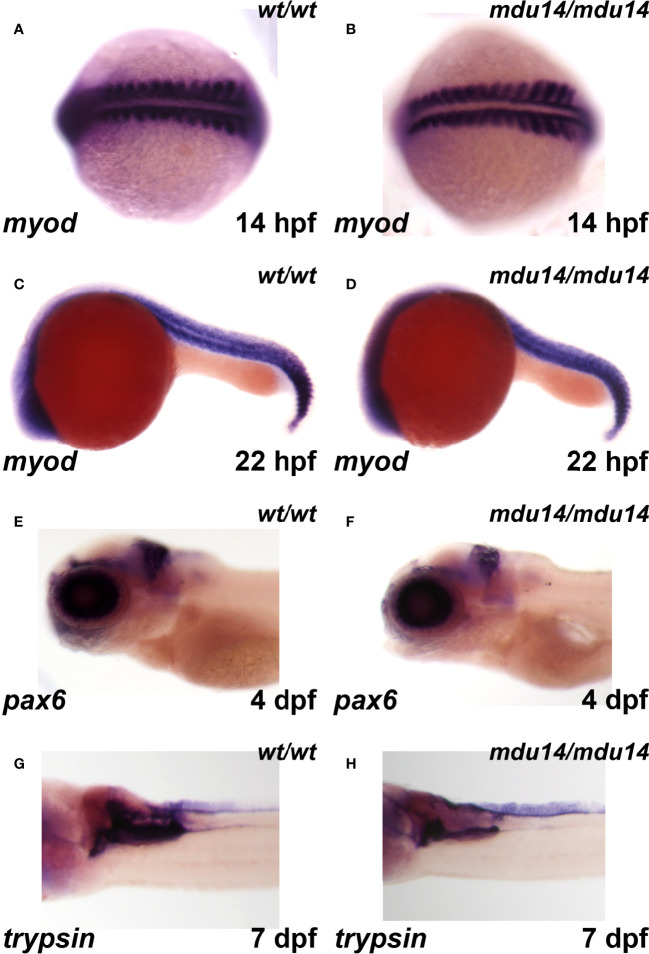
Mutation of *crlf3* fails to affect other relevant embryonic tissues. **(A–H)** Homozygous wild-type *crlf3^wt/wt^* (*wt/wt*), and *crlf3^mdu14/mdu14^* (*mdu14/mdu14*) embryos at the indicated ages were subjected to WISH with *myod* **(A–D)**, *pax6* **(E, F)** and *trypsin* **(G, H)**, presented as dorsal view **(A, B)**, lateral view **(C–F)** or ventral view **(G, H)** with anterior to the left in each case.

Given the embryonic expression pattern of *crlf3*, the expression of markers specific for somites, eye, brain and pancreas were also examined. No difference in expression between *crlf3^mdu14/mdu14^* mutant and wild-type embryos was observed for *myod*, a marker of muscle lineages (46), at 14 hpf (**Figures 5A, B**) and 22 hpf (**Figures 5C, D**), *pax6*, a marker for retinal progenitor cells and ganglion cells in the eye and brain, respectively (47), at 5 dpf (**Figures 5E, F**), and *trypsin*, a marker of the exocrine pancreas (48) at 7 dpf (**Figures 5G, H**).

## Discussion

Cytokines and cytokine receptors play a vital role in cell-to-cell communication important for hematopoiesis, immunity and other homeostatic processes (1). A defining characteristic of cytokine receptors is the presence of a conserved cytokine receptor homology domain (CHD) in the extracellular region. CRLF3 contains a CHD (3) that likely represents the archetypal CHD from which those found in all cytokine receptors were derived (5), with homologues of CRLF3 identified in a wide range of metazoan species (3). However, the function of this evolutionarily important protein remains poorly defined. This study used zebrafish as model to add to our understanding of CRLF3 in early development.

Zebrafish *crlf3* was found to be expressed at several hematopoietic sites during embryogenesis. This included the ALM, PLM and ICM, sites of early myelopoiesis, as well the thymus, location of T lymphocyte development, the latter consistent with data from mouse embryos (6). *Crlf3* has also been found to be expressed in the adult *Xenopus* thymus (49), adult mouse thymus and spleen (6) and adult human thymus, spleen and bone marrow (7). The zebrafish *crlf3* gene was additionally expressed in a range of other embryonic tissues, including adaxial cells within the pre-somitic mesoderm, regions of the developing eye and brain, as well as the pancreas. Broad expression has also been observed in mouse embryos, with *Crlf3* expressed in the mid-brain, sensory organs, ovary, testis, liver and other visceral organs (6). In adult *Xenopus*, *crlf3* was expressed in the central nervous system and testis (49), with mammals also showing expression in the central nervous system, liver and a variety of other sites (6, 7). This collectively suggests potential roles throughout the life-course in blood and immune cells, where cytokine receptors play vital roles (2), as well as more broadly across other tissues.

Genome editing was used to generate two mutants of zebrafish *crlf3*. These most likely represent loss-of-function alleles since they only encoded 7 or 8 residues of the 444 amino acids comprising the CRLF3 protein (<2% of the total). Moreover, heterozygotes of either mutant produced no discernible phenotype (data not shown). Therefore, homozygous *crlf3* mutants were analyzed with respect to early hematopoiesis in comparison to wild-type individuals. During the primitive wave of hematopoiesis, the *crlf3* mutants showed no alterations in the expression of markers for bipotential hemangioblast cells or vascular precursors, but those for hematopoietic, myeloid and erythroid progenitors were reduced, as were markers for mature myeloid and erythrocytic cells. Collectively, these results indicate *crlf3* likely impacts primitive hematopoiesis at the level of hematopoietic progenitors that affects downstream lineages. During definitive hematopoiesis, the markers for HSCs, mature myeloid and erythroid cells remained reduced, as did those for mature T lymphocytes – although not lymphocyte precursors. This suggests a broad role for *crlf3* in definitive hematopoiesis, affecting HSCs and their downstream lineages as well as the production of mature lymphocytes. In each case, however, the impacts were relatively modest indicating it is not an essential regulator.

There is some evidence from other studies that CRLF3 contributes to blood and immune cell development and/or function throughout the life-course. Human *CRLF3* variants have been associated with lymphocyte percentage in the blood (50) and risk of cutaneous leishmaniasis (51), while variants in the corresponding chicken gene are associated with an altered antibody response (52). It also forms part of a rare *UTP6-CRLF3* fusion observed in human acute myeloid leukemia (53). Ablation in mice caused reduced platelet numbers due to a defect in platelet maturation, and consistent with an association between human *CRLF3* variants and platelet distribution (50). *Crlf3^−/−^* mice also exhibited altered red blood cell parameters but no significant differences in immune cell populations or overall survival (54, 55). These observations are collectively consistent with a subtle role for CRLF3 in the hematopoietic system.

The broad embryonic expression of *crlf3* promoted the analysis of other tissues. However, the expression of key markers of skeletal muscle, retinal progenitor cells and ganglion and exocrine pancreas were unaltered. Other studies have suggested roles in neuronal differentiation and function (10, 56), with a *CRLF3* variant associated with the extent of autism in NF1 patients (56). On-line data on the *Crlf3^−/−^* mouse have also suggested reduced body weight and abnormal behaviors and tremors (55). However, no obvious differences in behavior or growth were observed in the *crlf3* mutants (data not shown).

The mechanism of action for CRLF3 remains vague, although there is high conservation of the protein across bilaterians, including vertebrates and insects (**Supplementary Figure 2**). This is particular true in the CHD that incorporates the archetypal WSXWS domain, but also extends to residues in the C-terminus, suggesting both are important for function. Indeed, evidence from insect systems suggest that CRLF3 is activated by cytokines, with both exogenous mammalian erythropoietin and an as yet unidentified endogenous cytokine in the hemolymph able to elicit CRLF3-dependent responses (57), suggesting the CHD is functional in cytokine binding. The insect CRLF3 has also been shown to lie upstream of the JAK-STAT pathway (8), while mammalian studies have also identified STAT3 as a downstream mediator (58), actions presumably mediated by the C-terminus of the protein. In the latter context CRLF3 has been implicated in cell growth as a result of concomitant elevation of cyclin D proteins and NF-κb (58). This is consistent with the upregulation of *CRLF3* observed during the early cancerous stage of actinic keratosis, cutaneous squamous cell carcinoma and non-melanoma skin cancer (59), and its involvement in the *UTP6-CRLF3* fusion in acute myeloid leukemia (53). However, a distinct role is also emerging in the regulation of cell and organelle morphogenesis, with its ablation impacting neurite outgrowth (56), synaptic vesicle biogenesis (10) and the maturation of platelets from large pre-platelet precursors (50), while its enforced expression perturbed cell morphology and motility (58). These effects have been associated with altered microtubule stability (50) and disrupted RhoA signaling (56, 58), with the Hippo pathway also implicated (50). It is hoped that the *crlf3* mutant will allow the further exploration of CRLF3’s mode of action.

## Data Availability Statement

The original contributions presented in the study are included in the article/**Supplementary Material**. Further inquiries can be directed to the corresponding author.

## Ethics Statement

The animal study was reviewed and approved by Deakin University Animal Ethics Committee.

## Author Contributions

TT, KP and CL performed experiments; TT, KP, AW and CL analyzed the results and prepared figures; YG, AW and CL designed the research; TT, KP, AW and CL wrote the paper, which was read and approved by all authors.

## Funding

The authors recognize the support of funding from IMPACT at Deakin University. TT was supported by a Deakin University International Postgraduate Research Award.

## Conflict of Interest

The authors declare that the research was conducted in the absence of any commercial or financial relationships that could be construed as a potential conflict of interest.

## Publisher’s Note

All claims expressed in this article are solely those of the authors and do not necessarily represent those of their affiliated organizations, or those of the publisher, the editors and the reviewers. Any product that may be evaluated in this article, or claim that may be made by its manufacturer, is not guaranteed or endorsed by the publisher.
