# Supplementary material for: Cytokine Receptor-Like Factor 3 (CRLF3) Contributes to Early Zebrafish Hematopoiesis
Source: Front Immunol. 2022 Jun 20;13:910428. doi: 10.3389/fimmu.2022.910428 (PMC9251315; doi:10.3389/fimmu.2022.910428)

Supp. Figure 1: Alternate allele of *crlf3* causes similar effects on primitive and early definitive hematopoiesis. Homozygous $crlf3^{wt/wt}$ (*wt/wt*) and $crlf3^{mdu15/mdu15}$ (*mdu15/mdu15*) embryos were subjected to WISH at 20 hpf with *ikzf1* **(A-B)**, at 4 dpf with *cmyb* **(G-H)** and at 22 hpf and 5 dpf for the remaining markers, as indicated. Individual embryos were assessed for area of staining *ikzf1* at 20 hpf **(C)**, *hbbe* at 22 hpf **(F)** and 5 dpf **(L)**, or the number of $lcp1^+$ cells at 22 hpf **(D)** and 5 dpf **(J)** or mpo^+ cells at 22 hpf **(E)** and 5 dpf **(K)**, with the mean and SEM shown in red and level of statistical-significance indicated (*** p<0.001, ** p<0.01, * p<0.05).

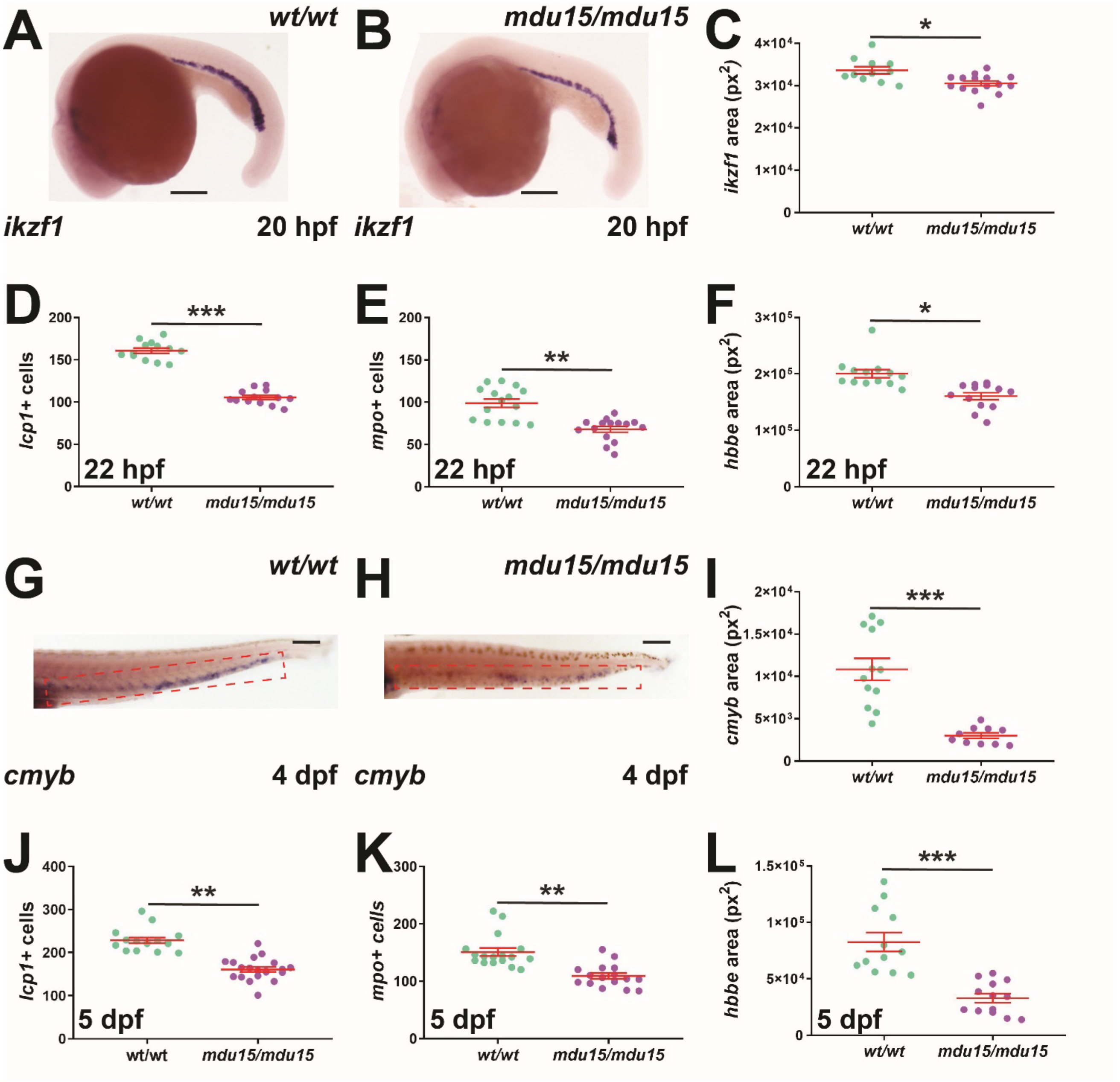

Supplement: Supplementary file 1 [file Image_1.pdf]
