# Supplementary material for: Cytokine Receptor-Like Factor 3 (CRLF3) Contributes to Early Zebrafish Hematopoiesis
Source: Front Immunol. 2022 Jun 20;13:910428. doi: 10.3389/fimmu.2022.910428 (PMC9251315; doi:10.3389/fimmu.2022.910428)

Supp. Figure 2: Multiple sequence alignment of CRLF3 protein sequences. (A) Multiple sequence alignment of CRLF3 sequences from human (hs – *Homo sapiens*), mouse (mm – *Mus musculus*), chicken (gg – *Gallus gallus*), xenopus (xl – *Xenopus laevis*), zebrafish (dr – *Danio rerio*) and locust (lm – *Locusta migratoria*). Amino acids in the multiple sequence alignment are denoted as identical (*), very similar (:) or similar (.). **(B)** CRLF3 protein identity matrix between species. **(C)** A consensus Neighbour-Joining tree of CRLF3 sequences using the locust sequence as the outgroup and 1000 replicates (indicated), with branch lengths shown.

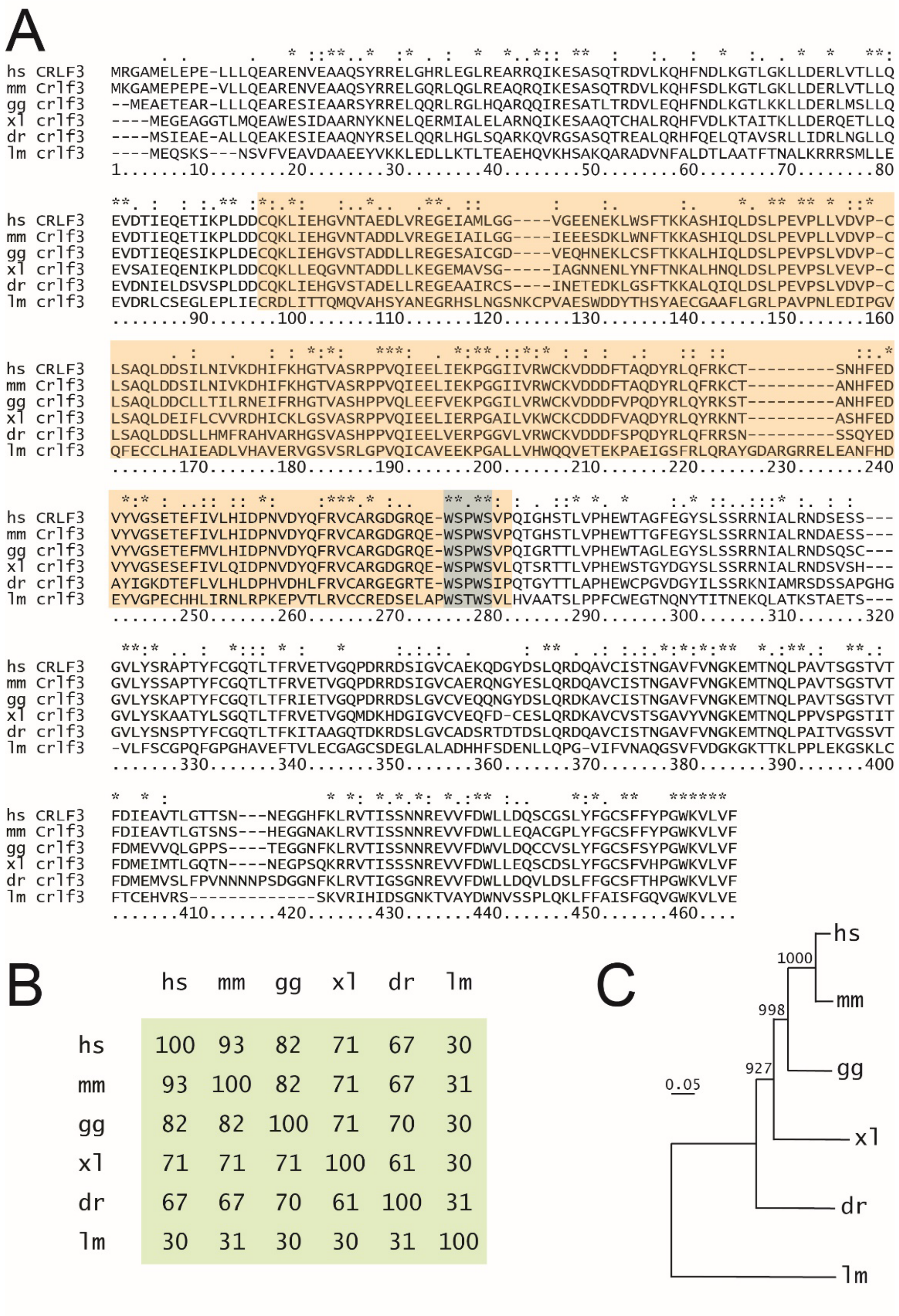

Supplement: Supplementary file 2 [file Image_2.pdf]
